# TRPV1 Marks Synaptic Segregation of Multiple Convergent Afferents at the Rat Medial Solitary Tract Nucleus

**DOI:** 10.1371/journal.pone.0025015

**Published:** 2011-09-20

**Authors:** James H. Peters, Stuart J. McDougall, Jessica A. Fawley, Michael C. Andresen

**Affiliations:** Department of Physiology and Pharmacology, Oregon Health & Science University, Portland, Oregon, United States of America; The Research Center of Neurobiology-Neurophysiology of Marseille, France

## Abstract

TRPV1 receptors are expressed on most but not all central terminals of cranial visceral afferents in the caudal solitary tract nucleus (NTS). TRPV1 is associated with unmyelinated C-fiber afferents. Both TRPV1+ and TRPV1- afferents enter NTS but their precise organization remains poorly understood. In horizontal brainstem slices, we activated solitary tract (ST) afferents and recorded ST-evoked glutamatergic excitatory synaptic currents (ST-EPSCs) under whole cell voltage clamp conditions from neurons of the medial subnucleus. Electrical shocks to the ST produced fixed latency EPSCs (jitter<200 µs) that identified direct ST afferent innervation. Graded increases in shock intensity often recruited more than one ST afferent and ST-EPSCs had consistent threshold intensity, latency to onset, and unique EPSC waveforms that characterized each unitary ST afferent contact. The TRPV1 agonist capsaicin (100 nM) blocked the evoked TRPV1+ ST-EPSCs and defined them as either TRPV1+ or TRPV1- inputs. No partial responses to capsaicin were observed so that in NTS neurons that received one or multiple (2–5) direct ST afferent inputs – all were either blocked by capsaicin or were unaltered. Since TRPV1 mediates asynchronous release following TRPV1+ ST-evoked EPSCs, we likewise found that recruiting more than one ST afferent further augmented the asynchronous response and was eliminated by capsaicin. Thus, TRPV1+ and TRPV1- afferents are completely segregated to separate NTS neurons. As a result, the TRPV1 receptor augments glutamate release only within unmyelinated afferent pathways in caudal medial NTS and our work indicates a complete separation of C-type from A-type afferent information at these first central neurons.

## Introduction

The primary viscerosensory nucleus of the medulla is the solitary tract nucleus (NTS) providing the initial stages of reflex control of homeostasis across multiple vital organ systems [1], [2]. Visceral afferents from several cranial nerves form the solitary tract (ST) and converge on the caudal NTS. Anatomical studies using cranial nerve tracing suggest considerable overlap across viscerotopic afferent maps [1], [3], [4]. In caudal NTS, the arbors of single second order neurons commonly reach considerable distances and likely support sizable targets for ST afferent contacts [4]–[7]. Cranial primary afferents are broadly diverse in their tissue origin and specific phenotypes (e.g. end-organ innervation, modality, neuropeptide expression) [8]–[10]. In horizontal slices, previous intracellular studies demonstrate that medial NTS neurons receive unitary excitatory postsynaptic currents (EPSCs) activated at specific threshold shock intensities with invariant latency and EPSC waveforms [11]–[13]. Recent studies have provided evidence that some NTS neurons are innervated by more than one ST afferent input [14], [15]; however, little is known about the nature or patterns of convergence of ST afferent synaptic contacts in NTS.

Visceral afferents within cranial nerves are remarkably heterogeneous, but as with all primary sensory neurons, they can be subdivided broadly into myelinated and unmyelinated phenotypes. As with spinal sensory afferents [16], [17], the “transient receptor potential vanilloid subtype 1” (TRPV1) ion channel is selectively expressed in craniovisceral primary sensory neurons with unmyelinated (C-type) axons but generally not expressed on myelinated fibers (A-type) [13]. Application of the TRPV1 agonist capsaicin at high concentration (100 nM CAP) completely blocked the ST-evoked EPSCs from some afferents (TRPV1+) but not others (TRPV1−) [13], [18]. Not only is TRPV1 a discriminating marker across these subclasses, but activation of TRPV1+ ST afferents promotes a surge of additional “asynchronous” glutamate release that trails for many seconds following cessation of ST stimulation [19]. These asynchronous EPSCs depended on activation of TRPV1 channels and occurred in all TRPV1+ neurons [19]. A key question is whether TRPV1+ and TRPV1− ST afferents can converge onto the same second-order NTS neurons.

In the present study, we identified second order NTS neurons receiving TRPV1+ and TPRV1− ST EPSCs in horizontal brainstem slices. Using recruitment profiles of the EPSC responses to graded-intensity ST shocks, we systematically characterized multiple discrete primary afferent inputs under voltage clamp and determined their sensitivity to CAP. Recruitment of additional ST afferents resulted in a summing of EPSCs into larger compound events. With each added ST-EPSC input, trains of ST shocks produced added increments in the magnitude of asynchronous release as well. In all cases, both synchronous and the associated asynchronous EPSC activity was either completely sensitive or resistant to CAP. Thus, second order medial NTS neurons receive multiple inputs through a single chemical class of afferents based on TRPV1 expression – a result that suggests that myelinated and unmyelinated afferents are fully segregated in the medial subnucleus of caudal NTS.

## Materials and Methods

### Animals

All animal procedures were performed with the approval of the Institutional Animal Care and Use Committees at Oregon Health & Science University (Portland, Oregon, USA) Protocol #IS00000578 in accordance with the United States Public Health Service Policy on Humane Care and Use of Laboratory Animals and National Institutes of Health Guide for the Care and Use of Laboratory Animals. In all experiments adult male Sprague Dawley rats (>160 g) were utilized. Animals were housed under 12 h light/12 h dark conditions and fed standard pellet chow *ad libitum*.

### Horizontal brainstem slices

Brain stem slices were prepared from isoflurane anesthetized rats as previously described [13], [19]. Briefly, the medulla was removed and placed in cooled artificial cerebrospinal fluid (aCSF) containing (mM): 125 NaCl, 3 KCl, 1.2 KH_2_PO_4_, 1.2 MgSO_4_, 25 NaHCO_3_, 10 glucose, and 2 CaCl_2_ that was bubbled with 95% O_2_–5% CO_2_. The brain was trimmed to yield a tissue block centered on obex. A wedge of tissue was removed from the ventral surface so that a horizontal cut yielded a single 250 µm thick slice that contained the ST together with NTS neuronal cell bodies. Slices were cut with a sapphire knife (Delaware Diamond Knives, Wilmington, DE) mounted in a vibrating microtome (VT1000S; Leica Microsystems Inc., Bannockburn, IL). Slices were secured in a custom perfusion chamber with a fine polyethylene mesh (Siskiyou Design Instruments, Grants Pass, OR) and perfused with aCSF (300 mOsm) constantly bubbled with 95% O_2_–5% CO_2_ at 34°C.

### In vitro whole cell patch recordings

The anatomical landmarks preserved in the horizontal slices allowed targeting of neurons within the medial sub-nucleus of the caudal NTS. Recorded neurons were located medial to the ST and within 200 µm rostral or caudal of obex. Patch electrodes were visually guided to neurons using infrared illumination and differential interference contrast optics (DIC) (40× water immersion lens) on an Axioskop 2 microscope (Zeiss, Thornwood, NJ) with digital camera (Hamamatsu Photonic Systems, Bridgewater, NJ). Recording electrodes (2.8–3.5 MΩ) were filled with a low Cl^−^ (10 mM, E_Cl_ = −69 mV) intracellular solution containing (mM): 6 NaCl, 4 NaOH, 130 K-gluconate, 11 EGTA, 1 CaCl_2_, 1 MgCl_2_, 10 HEPES, 2 Na_2_ATP, and 0.2 Na_2_GTP. The intracellular solution was pH 7.3 and 296 mOsm. Neurons were studied under voltage clamp conditions with a MultiClamp 700B amplifier (Molecular Devices, Union City, CA) and held at V_H_ = −60 mV using pipettes in open, whole cell patch configuration. Signals were filtered at 10 kHz and sampled at 30 kHz using p-Clamp software (version 9.2, Molecular Devices). Liquid junction potentials were not corrected.

### In vitro identification of second-order NTS neurons

A concentric bipolar stimulating electrode (200 µm outer tip diameter; Frederick Haer Co., Bowdoinham, ME) was placed on distal portions of the visible ST rostral to the recording region. Current shocks were delivered to the ST every 6 s (shock duration 100 µs) using a Master-8 isolated programmable stimulator (A.M.P.I., Jerusalem, Israel). Suprathreshold shocks to the distal ST evoked fixed latency EPSCs [20]. Latency was measured as the time between the ST shock and the onset of the resulting EPSC. Synaptic jitter was calculated as the standard deviation of ST-EPSC latencies for 30–40 trials within each neuron. Jitters of <200 µs reliably identify direct, monosynaptic afferent inputs [20], [21].

### Analytic measures and statistical testing

Digitized waveforms were analyzed using an event detection and analysis program (MiniAnalysis, Synaptosoft, Decatur, GA) for all quantal synaptic currents and Clampfit 10 (Axon Instruments, Foster City, CA) for all ST evoked EPSCs. To assure accurately measured amplitudes and kinetics of quantal events, events smaller than 10 pA were excluded. To determine the number of additional events released asynchronously, the frequencies of quantal events were calculated for 100 ms bins and summed across 50 stimulation trials. The asynchronous rate was expressed as the net increase in events above the unstimulated baseline. Thus, the average rate of baseline events across all trials measured for 1 s before each shock train commenced was subtracted from the rate calculated for each 100 ms bin during the period of 4 s following the last ST shock to yield a net asynchronous event rate. Across trials, the average frequency of events during the first 1000 ms following the last ST stimulus shock was a measure of the ‘peak’ asynchronous frequency. For statistical comparisons T-tests, Mann Whitney Rank Sum test, and linear regression analysis were used when appropriate. (Sigma Stat, San Jose, CA). For group comparisons p<0.05 was considered statistically significant.

## Results

### Increases in shock intensity recruit multiple monosynaptic ST afferents

**Figure 1:**
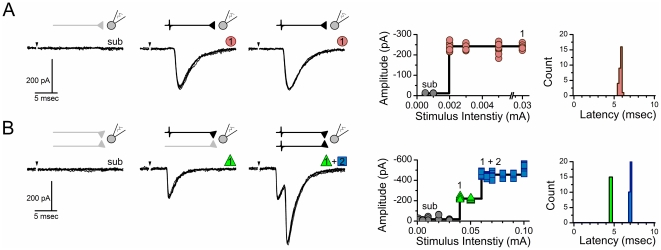
Incremental stimulus recruitment curves differentiate between single and multiple monosynaptic ST-NTS contacts. Increases in shock intensity to the ST triggered fixed latency glutamatergic EPSCs with added complexity of waveforms. The fixed latency is consistent with recruitment of added single afferent inputs. **A**) Representative neuron that received a single input that was unaffected by further increments in shock intensity consistent with a lack of convergent addition axons (model organization, above. The recruitment profile for such responses showed a single sharp intensity threshold and a plateau of constant amplitude despite substantial higher stimulus strength. The latency histogram showed a narrow band of values from which jitter is calculated. **B**) In other neurons, raising shock intensity activated additional synaptic responses and in this case this added EPSC arrived at a later time but with low jitter. The recruitment profile for such compound responses showed additional sharp intensity thresholds with narrow plateaus of constant amplitude for each convergent input. The equivalent pathway circuit suggest two parallel ST afferents converging on the same ST neuron. Note that thresholds were precise and ‘all-or-none’ over limited intensity ranges making each component response consistent with unitary contributions adding to the more complex EPSC.

Graded increases in the intensity of shocks applied to the ST detected the threshold for an afferent axon connected to the recorded neuron and a constant latency ST-EPSC appeared (Figure 1A). In some neurons, further increases in ST shock intensity failed to change the shape or amplitude of the EPSC (Figure 1A). The combination of a fixed latency that varied <200 µs (monosynaptic jitter) from shock to shock and the steady amplitude with increased shock intensity was consistent with a single ST afferent monosynaptically contacting the recorded neuron. In other neurons, increments in ST shock intensity recruited additional ST inputs. Multiple ST afferents converging on the same neuron were indicated by changes in the shape and/or amplitude of the now compound EPSC waveforms at the recorded neuron (Figure 1B). Within neurons, these added inputs appeared at different latencies and each monosynaptic event within the compound EPSC met the low jitter criterion for monosynaptic connections. Each input had a similarly narrow latency distribution (Figure 1, right panels). It is important to note that even closely adjacent neurons in medial NTS do not share common ST inputs [15]. In the present study (n = 27 neurons), recruitment curves indicated that roughly one third of second order neurons received a single ST input (n = 10, 37%) but most NTS neurons had more complex EPSC waveforms with added monosynaptic ST synaptic responses to increases in ST shock intensity (n = 17 of 27, 63%).

### Capsaicin divides neurons to TRP1+ and TRPV1− subtypes

**Figure 2:**
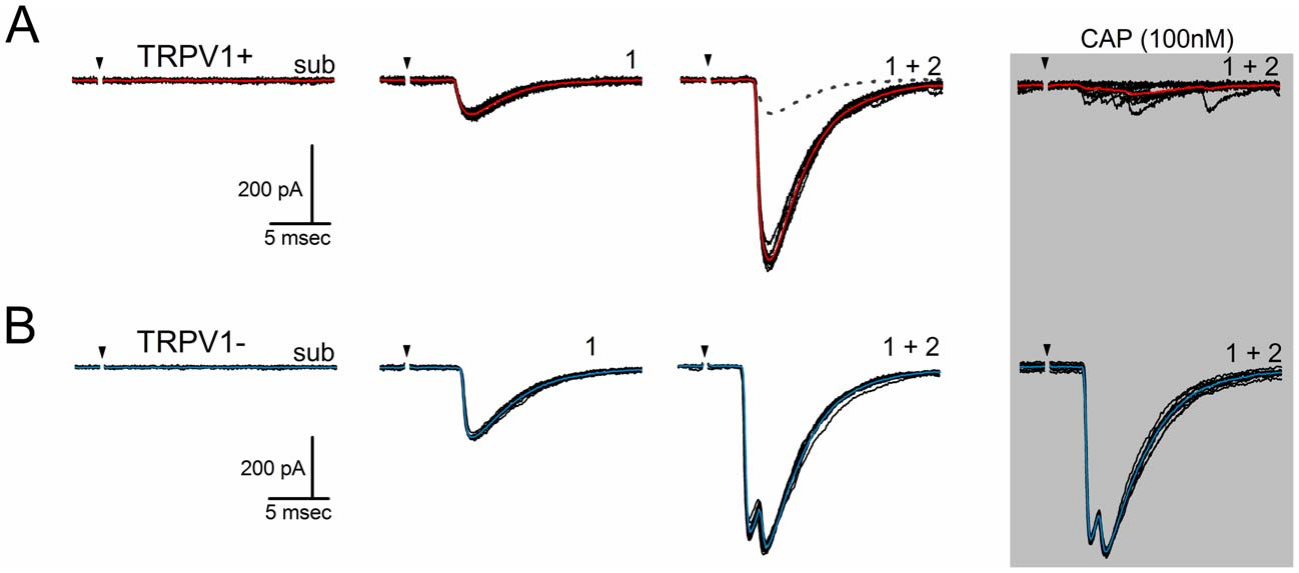
Convergent ST afferent inputs are either capsaicin sensitive or resistant. **A**) In most cases, increases in shocks to the ST recruited more than one afferent (1+2) to produce a compound EPSC (broken line depicts the low threshold EPSC 1 for comparison). Addition of the TRPV1 agonist capsaicin (100 nM) blocked the synchronous, high intensity compound EPSCs (1+2, gray shaded box). In such TRPV1+ neurons, CAP increased the number of baseline stochastic EPSCs despite blocking ST synced events. **B**) In other neurons, CAP had no effect on the synaptic response to ST activation of two convergent ST axons and thus was considered TRPV1−. Current traces are 5 sweeps superimposed, stimulus artifacts removed. The colored trace is the average waveform.

Following determination of the maximally effective ST shock intensity from the recruitment protocol, the TRPV1 agonist CAP (100 nM) was added to test for TRPV1 receptors. In most neurons (TRPV1+), CAP produced a blockade of ST-evoked EPSCs (Figure 2A). Note that this representative neuron had two distinctly different, monosynaptic inputs to high intensity ST shocks and both ST-synced EPSCs were blocked by CAP (Figure 2A). As previously reported [19], CAP substantially increased the rate of spontaneous EPSCs despite inhibition of the ST-synced EPSCs (Figure 2A). However in TRPV1− neurons, CAP had no effect on the amplitude of any of the ST-evoked EPSCs (Figure 2B). In both of these representative examples (Figure 2), each neuron received two separate, low jitter ST inputs and each of the component EPSCs was either blocked or CAP-resistant. Thus, CAP blockade was all-or-none across ST afferents at single neurons. On the basis of the CAP test, neurons with multiple inputs could be classified as either TRP1+ or TRPV1−. No cases of mixed responses were detected.

**Figure 3:**
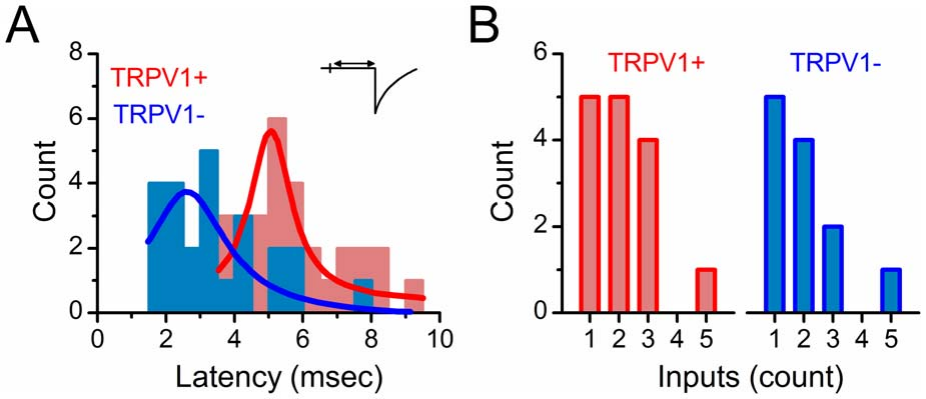
TRPV1+ and TRPV1− similarly converge on NTS neurons. Overall, TRPV1− inputs (blue) to NTS neurons had shorter latencies than TRPV1+ inputs (red). **A**) Histogram of latency to EPSC onset. TRPV1− afferent inputs (n = 24 inputs) arrived more quickly than TRPV1+ (n = 32 inputs). This result was consistent with afferent myelination associate with TRPV1− and faster conduction velocity although axonal distances for ST cannot be precisely determined. **B**) The number of detected ST inputs was similar between the two classes. Count is number of direct ST inputs measured. Number of monosynaptic ST afferent inputs onto NTS neurons. Single or multiple inputs were either completely blocked (n = 15 neurons, red bars, TRPV1+) or resistant (n = 12 neurons, blue bars, TRPV1−) to capsaicin (CAP). Count is number of recorded neurons.

Across this group of recorded neurons (n = 27, Figure 3), the TRPV1+ group consisted of 32 monosynaptic inputs across 15 neurons and were completely blocked by CAP. For the TRPV1− group, 24 monosynaptic inputs across 12 neurons were unaltered by CAP. The aggregate ST-EPSC latencies for these two groups were significantly shorter for TRPV1− than for TRPV1+ neurons (2.94±0.32 ms vs. 5.09±0.27 ms, p<0.001), although there was substantial overlap. The shorter latencies and CAP-resistance (Figure 3A) were consistent with faster conduction velocities presumed for myelinated axons. However, conduction velocities could not be accurately assessed since the length of the ST fiber to the neuron was poorly defined. The absolute number of detected afferents was relatively limited and the majority of neurons received 1–3 ST monosynaptic inputs with similar distributions between TRPV1+ and TRPV1− afferents (Mann-Whitney Rank Sum test, p = 0.7) (Figure 3B). The mean ST-EPSC amplitudes of the first recruited event were similar (p>0.05) between TRPV1− inputs (−245.3±80.9 pA, n = 15) and TRPV1+ (−169.7±23.8 pA, n = 12).

### Frequency dependent depression is unaffected by multiple inputs

**Figure 4:**
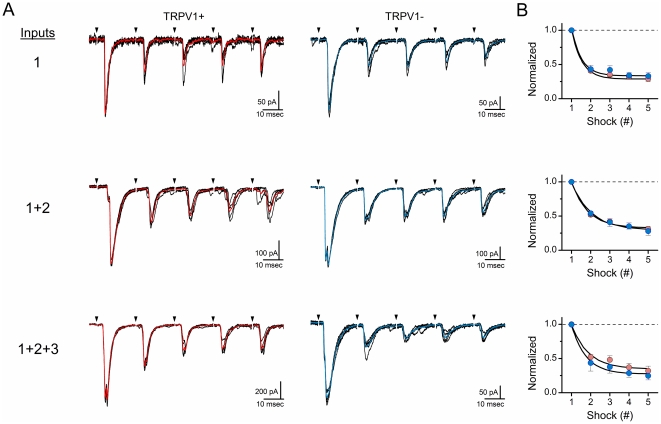
Frequency dependent depression of ST-EPSCs was similar regardless of the number of afferent inputs. **A**) Multiple examples from different neurons representing synaptic depression in neurons with one to three monosynaptic ST inputs. Responses were to trains of ST shocks (5 shocks at 50 Hz) and shocks were maximally effective determined by recruitment protocols (see Figure 1 and text). The left column shows three different TRPV1+ (red) neurons and the right column shows three representative TRPV1− (blue) NTS neurons. **B**) Normalized curves for frequency dependent depression across NTS neurons with one activated input (TRPV1+ n = 15 and TRPV1− n = 12; top panel), two activated inputs (TRPV1+ n = 10 and TRPV1− n = 7; middle panel), or three activated inputs (TRPV1+ n = 5 and TRPV1− n = 3; bottom panel). In some cases, neurons with two or three inputs were studied at different stimulus intensities. In this manner, submaximal responses were included in particular groups, e.g. “one input activated” by measuring the events and their depression with the shock intensity set to a level that activated only one or two of the three ST inputs found at high intensities. Mean values from TRPV1+ afferents are indicated in red and TRPV1− are indicated in blue. Points are mean ± SEM normalized to EPSC1. ANOVA found no differences in depression related to afferent class (TRPV1+/−) or numbers of inputs. Only shock number altered relative EPSC amplitude, i.e. frequency dependent depression (see text). Values are means±SEM.

Activation of single ST axons at high frequencies leads to characteristically substantial frequency dependent depression (FDD) [19], [20]. Here we asked whether FDD was changed when multiple afferents to the same neuron were activated. In each case, intensity-recruitment profiling defined both the number of monosynaptic ST axons to each neuron and the adequate suprathreshold shock intensity to activate them. The ST was then activated in bursts using suprathreshold intensity shocks (5 shocks at 50 Hz every 6 s). This 50 Hz burst was selected as physiologically relevant based on afferent arterial baroreceptor recordings [11], [22]. In neurons with multiple ST inputs, shock intensity was set to activate one, then two and then three so that these responses could be analyzed by the number of active inputs pooled across neurons (Figure 4). In all cases, the amplitude of the ST-EPSC was approximately 50% depressed by the second shock (Figure 4A). No differences in FDD were detected between TRPV1+ and TRPV1− neurons (ANOVA, p = 0.15). Further, comparison of FDD from neurons with single inputs, double inputs or triple inputs indicated no statistically differences so that degree of depression was found to be independent of the number of inputs activated (ANOVA, p = 0.14) (Figure 4B). Thus, recruitment of multiple ST afferents to a single neuron does not materially alter the underlying unitary rates of vesicle release, the depression or the recovery from depressed release.

**Figure 5:**
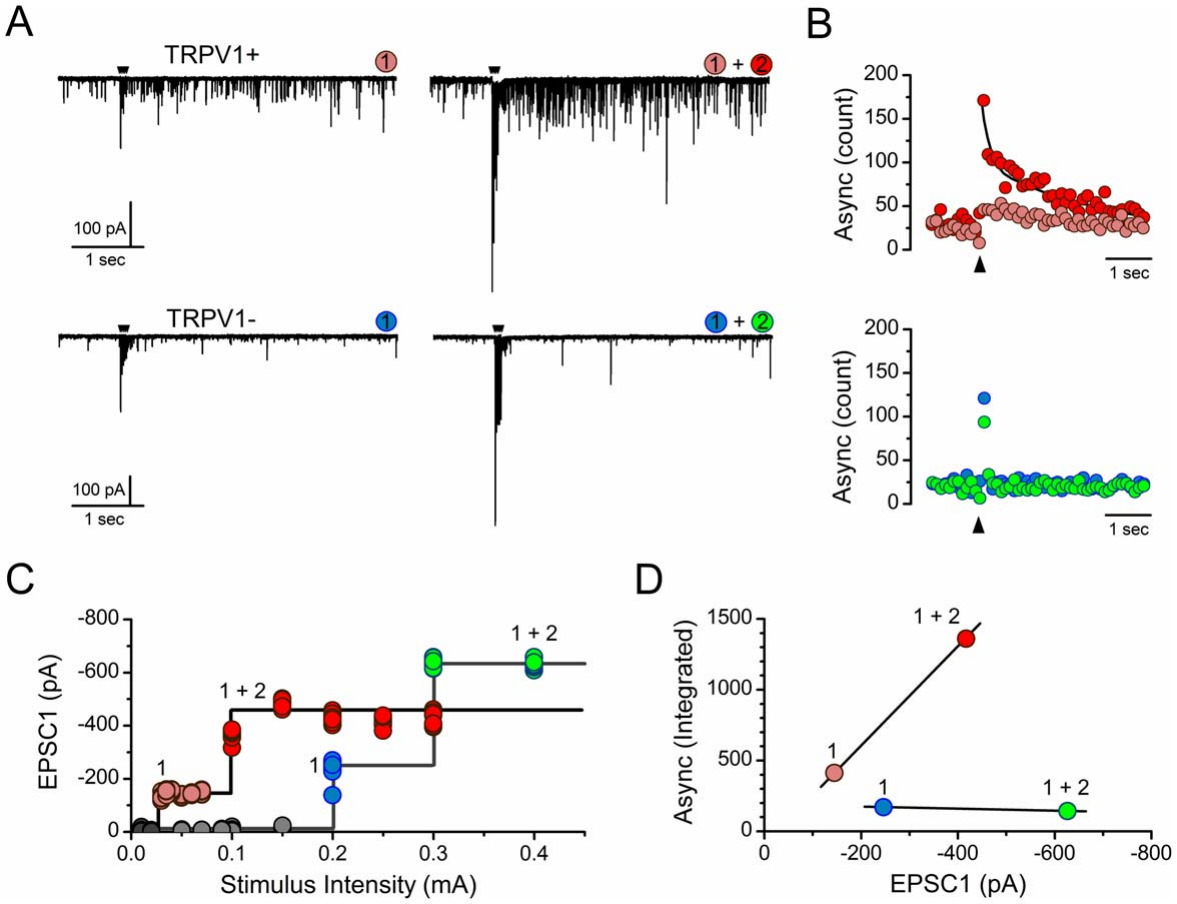
Recruitment of multiple TRPV1+ ST inputs produces added asynchronous glutamate release. **A**) Activation of TRPV1+ (representative neuron, upper traces, left) but not TRPV1− (different representative neuron, lower traces, left) ST inputs generates long lasting asynchronous glutamate release following the synchronous EPSCs. Increasing ST shock intensity recruited additional ST inputs and additional asynchronous release in TRPV1+ (upper traces, right) but not TRPV1− (lower traces, right). **B**) Counts of spontaneous quantal events prior to (basal) and asynchronous events following ST shocks (black arrow) showed that increments only occurred from TRPV1+ afferents (top, red). Added (1+2) recruitment of ST-EPSCs incremented the asynchronous TRPV1+ release. TRPV1− afferent (green) showed little activity in the asynchronous period and no effect of multiple ST inputs activation. **C**) Stimulus recruitment curves for synchronous EPSCs show detection of individual ST input thresholds and amplitude distributions. **D**) Recruitment of added ST-EPSCs augmented the integrated asynchronous release (additional events across 50 trials minus baseline frequency) for this representative TRPV1+ neuron; while no additional asynchronous release occurred with as large or larger multiple TPRV1− ST contacts in another representative neuron. Two neurons were same examples analyzed throughout A–D.

**Figure 6:**
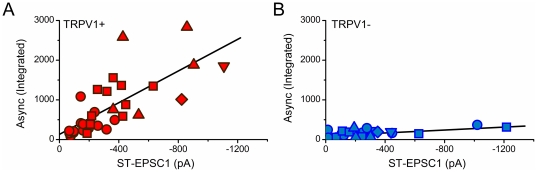
Overall asynchronous glutamate release increases with larger synchronous EPSC amplitudes in TRPV1+ but not in TRPV1− afferents. **A**) Across NTS neurons each additional input from TRPV1+ ST afferents (N = 15 neurons with n = 32 inputs) produced additional asynchronous release proportional to the evoked EPSC1. **B**. Additive TPRV1− inputs produced no additional asynchronous release (N = 12 neurons with n = 24 inputs). TRPV1+ inputs shown with red symbols and TRPV1− shown with blue symbols. Recruited inputs: 1^st^ = circle, 2^nd^ = square, 3^rd^ = triangle, 4^th^ = diamond, and 5^th^ = inverted triangle. Data are fit with a linear regression: TRPV1+ slope = 2.24±0.20, R^2^ = 0.55; and TRPV1− slope = 0.18±0.06, R^2^ = 0.30.

A diagnostic indicator of TRPV1+ ST contacts is the presence of a substantial asynchronous, stochastic release of glutamate following brief bursts of high frequency shocks to ST [19], [23]. Note that this occurs despite substantial FDD (Figure 5). In the present study, we found that CAP blocked all synchronous ST transmission, even in cases of multiple TRPV1+ ST inputs to single neurons. In this portion of our study, we asked whether the asynchronous release following bursts of ST activation was augmented by activating multiple ST fibers (Figure 5). Note that this asynchronous release was defined as the increase above the baseline rate of spontaneous EPSCs found before the ST shocks. The ST bundle was activated using bursts of suprathreshold shocks (5 shocks at 50 Hz every 6 s). Activation of single ST inputs evoked substantial asynchronous release in TRPV1+ neurons and increasing ST shock intensity recruited additional ST axons with augmented asynchronous activity as well (Figure 5). As expected, asynchronous activity was not evident in TRPV1− neurons whether one or multiple ST-EPSCs were recruited (Figure 5). Both the peak frequency and total number of additional quantal events over baseline were greater when multiple ST inputs were activated (Figure 5B, top). Activation of TRPV1− afferents produced no additional release even when multiple large inputs were recruited (Figure 5A and B, lower). Each afferent input had discrete thresholds of activation as detected by analysis of stimulus recruitment curves (Figure 5C). In aggregate across 15 neurons, increments in the amplitude of the compound ST-EPSC waveform by recruiting multiple ST afferents produced greater amounts of asynchronous release from TRPV1+ contacts (Figure 6A), but stochastic release was not changed for TRPV1− contacts despite similarly substantial increases in the synchronous ST-EPSC magnitude and numbers of ST afferents recruited (Figure 6B).

## Discussion

Much of the evidence localizing the central destinations of cranial visceral afferents within NTS was established through neuroanatomical tracers and/or functional mapping studies based on extracellular recordings or focal NTS interventions to evoke reflex responses [1], [10]. Comparisons of these viscerotopic maps suggest considerable overlap of nerves and related functional domains within caudal NTS. At the heart of the viscerotopic idea is the presumption that neurons in close proximity should share common functions and thus similar afferent inputs. These maps provide global associations but are limited by their lack of information about synapse localization – the sites at which afferents functionally connect to central neurons. These synapses are the critical cellular underpinnings of afferent transmission shaped by their locations and organization. Thus, although substantial heterogeneity across the afferent neurons is well established [18], [24], little is known about afferent central relations. In the present studies, we used an electrophysiological approach to discriminate single second-order viscerosensory neurons and activated the central nerve trunk of these cranial nerve afferents, the ST, to determine the nature of ST-NTS transmission. A major strategic goal was to take advantage of the presence of a major cellular marker at primary sensory afferent terminals, TRPV1 [13], [18], in order to ask whether TRPV1+ and TRPV1− afferents contacted the same medial NTS neurons. Our major findings indicate that ST afferents within medial NTS are organized distinctly differently from what is known for spinal primary sensory afferents. We found that at NTS second order neurons: 1. Monosynaptic ST afferents were either sensitive to the TRPV1 agonist CAP or not. 2. Despite multiple monosynaptic convergent ST afferents, TRPV1 classes (+/−) never mixed. 3. As with synchronous ST-EPSCs, multiple ST afferents contributed additional asynchronous glutamate release. 4. Multiple and single ST afferents depressed similarly regardless of TRPV1 class. Since the expression of TRPV1 is predictive of afferent phenotype (generally C-fiber) [18], our results and others [25] indicate a strict separation of information across this cellular dichotomy. Thus, at the level of the first central synapses, these medial NTS neurons are either C- or A-fiber receptive.

### Identifying discrete ST afferent inputs

A critical and informative technical aspect in our experimental design was to distinguish between directly evoked monosynaptic EPSC pathways and other, indirectly arriving glutamatergic inputs that arise from more complicated routes (polysynaptic pathways) [26]. This aspect of transmission is critical to discern for two reasons: 1. ST afferents are synaptically organized and activated differently from central excitatory inputs. 2. The glutamate release mechanisms for ST primary afferents and central excitatory inputs are distinct [14], [26]. In particular, TRPV1 [27] is generally restricted to primary sensory afferents [28]. In caudal NTS, higher order EPSCs are substantially smaller and less reliable than ST-EPSCs [14], [26]. The synaptic contacts of viscerosensory afferent terminals on second order neurons within medial NTS have several surprising properties compared to other sensory and central neuron systems [29]. The ST delivery of glutamate generates remarkably reliable EPSCs with a large safety factor for signaling and in part this results in the faithful generation of postsynaptic spiking despite substantial FDD, [20], [30]. Previous studies indicate that despite the diversity of cranial sensory neurons, the basic glutamate release scheme for TRPV1+ and TRPV1− afferents was quantitatively comparable in quantal content, number of active release sites, release probability and calcium sensitivity [31]–[33] and/or between anatomically identified aortic baroreceptor compared to non-baroreceptor afferents [33]. Thus, synchronous release triggered by incoming action potentials is remarkably similar in substantial mechanistic detail despite the overt heterogeneities in cellular phenotype. As in our previous studies [15], the neuron groups in the present study had similar numbers of monosynaptic inputs and mean amplitudes. Even *in vivo*, responses to synaptic activation from the carotid sinus and laryngeal nerve trunks were similar and that included both A- and C-fiber afferents [34]. In studies of spontaneous events recorded from neurons within the centralis subnucleus of NTS [35], a mixed organization of pharmacologically defined inputs raises the possibility that different organizational patterns may prevail in different subnuclei across NTS.

### Asynchronous release marks TRPV1+ afferents

Synaptic efficacy is fundamentally determined by the strength of the underlying responses arising from single inputs (e.g. probability of release, quantal size, number of release sites), their location on the neuron relative to the spike initiation zone, and the total number of inputs converging onto an individual neuron [29]. Central neurons, such as those in the cortex, often receive large numbers (>50) of weak (near quantal), unreliable, individual synaptic inputs that arrive at distal dendrites and produce limited individual responses [36]. This configuration requires the synchronized coincident arrival of substantial numbers of convergent synaptic inputs in order to generate postsynaptic firing [36]. In contrast, second-order NTS neurons receive contacts originating often from one or two ST afferents which faithfully entrain the postsynaptic neuron [20]. The ST-NTS synapse achieves this high fidelity of transmission through multiple release sites concentrated within the proximal somatodendritic portion of the neuron [31]–[33] and this synaptic arrangement likely contributes to the remarkably unfailing afferent success in driving postsynaptic spikes – resulting in a high safety factor for afferent to neuron communication [20].

A fundamental distinction between TRPV1+ and TRPV1− ST transmission was asynchronous release. Despite the remarkable and detailed similarities in the glutamate release characteristics of synchronous transmission for TRPV1+ and TRPV1− ST afferents [19], [33], CAP sensitivity perfectly matched the incidence of asynchronous release. Individual synchronous EPSCs were blocked by CAP and the transient augmentation of stochastic EPSC activity following bursts of ST shocks also disappeared. Our graded recruitment protocols revealed that more intense shocks recruited multiple, low-jitter EPSCs in many neurons and that with each additionally recruited ST input there was an added asynchronous release for TRPV1+ ST afferents. Asynchronous release was not found with TRPV1− ST afferent activation regardless of the evoked EPSC amplitude or the number of ST inputs. Thus, asynchronous release is predictive of CAP sensitivity and vice versa. Functionally, the convergence of multiple TRPV1+ afferents onto single medial NTS neurons predicts that TRPV1-mediated plasticity [19] will amplify and extend afferent driven activity in proportion to the population of convergent TRPV1+ terminals activated. Future work needs to examine the impact on C-type reflex responses directly but it is tempting to speculate that this activity-dependent asynchronous enhancement of ST transmission contributes to the enhanced reflex efficacy of unmyelinated afferent baroreceptors concentrated in this medial region of NTS [37], [38].

The TRPV1 channel is active at normal temperatures and appears to sustain elevated spontaneous glutamate release from these afferent terminals compared to neurons receiving TRPV1− ST afferents in NTS [23]. Clearly, the presence of multiple TRPV1+ afferent axons contributing large numbers of contacting terminals provides a substantial ongoing release of glutamate. This ongoing release requires no action potential drive from the peripheral afferent sensory endings, endings whose peripheral neurons are most often physiologically silent under normal conditions. Despite this, such spontaneous EPSCs drive the postsynaptic neuron to discharge [23]. So beyond its potential role to boost the impact of afferent driven activity, this autonomous spontaneous EPSC activity may be important for other functions. Spontaneously released glutamate “miniatures” in other central pathways affect gene expression and maintain critical features of specialization of the postsynaptic neuron [39], [40]. This may be an important feature of the NTS since the unmyelinated class of craniovisceral primary sensory afferents dominates (70–90%) the total information input to the brain [9], [41]–[43]. Nothing is known about this possibility of a trophic interaction between ST afferents and second order NTS neurons. In addition, the continuous release of neurotransmitter may serve a signaling role unique to TRPV1+ synapses since the process can be modulated by G-protein coupled receptors. In some respects, this TRPV1 driven spontaneous release might compensate for the paucity of afferent activity and provide better balance with glutamate release at myelinated afferents which are physiologically discharging in normal conditions.

### Conclusions

Synaptic transmission from viscerosensory afferents occurs through an asymmetrical combination of synchronous and stochastic glutamate release. The synchronous release process is similar across all of these afferents but spontaneous and asynchronous glutamate release occurs at substantial rates only from TRPV1+ terminals. The present work establishes that this separation of release characteristics is absolutely segregated within medial NTS and that TRPV1 dependent release is proportionally accentuated depending on the number of converging afferent axons. The complete segregation of ST afferent inputs based on TRPV1 expression defines the type of synaptic integration and provides an organizational principle for distribution of primary afferent information.
